# *Culicoides segnis* and *Culicoides pictipennis* Biting Midges (Diptera, Ceratopogonidae), New Reported Vectors of *Haemoproteus* Parasites

**DOI:** 10.3390/microorganisms10050898

**Published:** 2022-04-25

**Authors:** Rita Žiegytė, Rasa Bernotienė, Vaidas Palinauskas

**Affiliations:** Nature Research Centre, Akademijos 2, 08412 Vilnius, Lithuania; rasa.bernotiene@gamtc.lt

**Keywords:** *Culicoides*, *Haemoproteus*, new vector, microscopy, sporozoites, genetic lineage

## Abstract

As bloodsuckers of birds, *Culicoides* biting midges (Diptera, Ceratopogonidae) play an important role in the transmission of avian haemosporidian (*Haemoproteus*) parasites, which are prevalent in many bird populations and cause disease, pathology, or even mortality in their hosts. Information about the role of the various *Culicoides* species in the transmission of *Haemoproteus* parasites remains insufficient. This presents an obstacle for the better understanding of the epizootiology of haemoproteosis. The aim of this study was to determine new *Culicoides* species involved in the transmission of *Haemoproteus* parasites in the wild. Biting midges were collected using UV traps on the Curonian Spit, Lithuania. Only parous *Culicoides* females were investigated: they were identified and were diagnosed for the presence of *Haemoproteus* parasites using both microscopy and PCR-based methods. We collected and dissected 420 parous *Culicoides* females. PCR-based screening showed that 28 parous *Culicoides* biting midges were infected with avian *Haemoproteus* parasites. Haemoproteid DNA was detected in *Culicoides kibunensis*, *Culicoides pictipennis*, *Culicoides festivipennis*, *Culicoides segnis*, *Culicoides pallidicornis,* and *Culicoides obsoletus* biting midges. The DNA of *Haemoproteus* *palloris*, genetic lineage hWW1, was found for the first time in *C. pallidicornis*. *Haemoproteus* sporozoites were detected in the salivary glands of two *Culicoides segnis* biting midges. According to the PCR results, one female contained *Haemoproteus tartakovskyi* (genetic lineage hHAWF1) DNA and another *Haemoproteus majoris* (genetic lineage hCCF5) DNA. The sporozoites of *Haemoproteus* parasites were also detected in the salivary glands of four *C. pictipennis* biting midges using microscopy, and this finding was confirmed by PCR as *Haemoproteus parabelopolskyi* DNA (genetic lineage hSYAT02) was detected in three out of the four biting midges. The obtained results supplement existing information about *Culicoides* biting midges as natural vectors of *Haemoproteus* spp. and add two new *Culicoides* species to the vector list, showing the low specificity of these parasites for the invertebrate hosts.

## 1. Introduction

*Culicoides* biting midges (Diptera, Ceratopogonidae) are widespread across almost all the world [1,2,3]. These blood sucking insects are known as vectors of avian haemosporidian parasites belonging to the genus *Haemoproteus* (Haemosporida, Haemoproteidae) [4,5,6,7,8,9]. The asexual reproduction of these parasites occurs in birds, which are intermediate hosts, while sexual reproduction takes place in biting midges [5,10]. *Haemoproteus* parasites can cause disease and even lethal pathology in vertebrate hosts, especially in non-adapted birds [11,12,13,14,15,16,17,18]. *Haemoproteus* infections are also virulent to some blood-sucking dipterans and can even kill the insects [19,20]. In order to understand the epizootiology and peculiarities of the transmission of these harmful parasites in the wild, it is of great importance to know the natural vectors of haemoproteids. From around 1400 described *Culicoides* species [3], only four species have been identified as natural vectors of haemoproteids in Europe [9,21], and ten more *Culicoides* species are known as vectors of *Haemoproteus* spp. worldwide [6]. In recent years, molecular methods have mostly been applied to test for infections of blood-sucking insects with *Haemoproteus* spp. [9]. However, it has been shown that avian haemosporidians can persist both in competent vectors and in resistant blood-sucking insects for several weeks after the initial infected blood meals [22]. PCR-based diagnostics cannot distinguish between sporozoites (the infective stage to vertebrate hosts) and non-infective sporogonic stages; therefore, this method alone cannot be used for detecting vectors of haemosporidian parasites. However, the presence of haemosporidian parasite DNA in blood-sucking insects, detected using solely PCR-based methods, provides new information on the feeding preference of biting midges, as these parasites can be gained only while feeding on birds [23] and also allows the identification of potential vectors of haemosporidian parasites. It is necessary to emphasize that microscopy and the detection of sporozoites is an important part in the detection of haemosporidian parasite vectors.

The aim of our study was to identify *Culicoides* species as vectors of *Haemoproteus* spp. in the wild and expand existing knowledge regarding the transmission of these parasites. We collected biting midges using UV traps, identifying them, dissecting individually each parous female for salivary gland preparations, and applying PCR-based analysis in order to determine if the insect had been infected with *Haemoproteus* parasites. The results of this study supplemented the list of *Culicoides* species known as vectors of haemoproteids in Europe by two *Culicoides* species.

## 2. Materials and Methods

### 2.1. Study Site, Collection of Biting Midges, and Preparation of Specimens for Microscopic Examination

**Figure 1:**
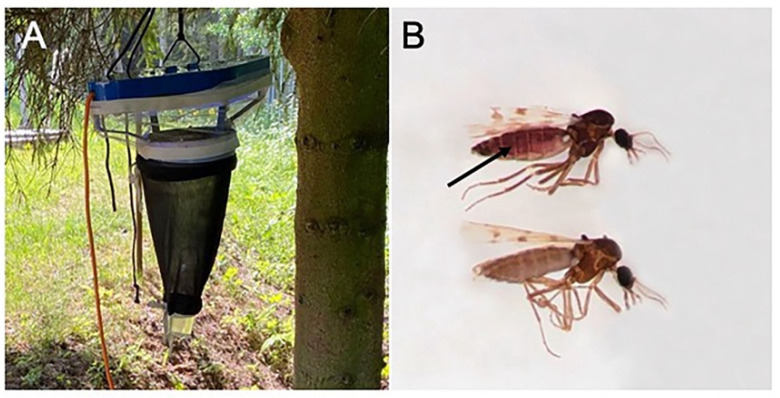
The UV trap in the forest for the catching of biting midges (**A**); parous (arrow) and nulliparous females based on the pigment coloration on the abdomen (**B**).

Biting midges were collected in June 2020 using an Onderstepoort 220 V UV trap close to Juodkrantė village (55.55676 N, 21.12398 E) on the Curonian Spit located by the Baltic Sea, Lithuania. The trap was hung in a swampy old forest dominated by spruce and alder; it was turned on 1–2 h before sunset and was turned off 2–3 h after sunrise (Figure 1A). Insects were collected in a water container supplemented with a drop of liquid soap as described by Bernotienė et al. [24]. Collected insects were transported to the laboratory of the Biological Station of the Nature Research Centre (Juodkrantė). Parous biting midge females were sorted by the burgundy pigment on their abdomens (Figure 1B) as reported by Dyce [25] and identified by their wing coloration and other morphological features [26,27,28]. The material was studied under binocular stereoscopic microscopes.

### 2.2. Microscopic Examination and Morphometric Analysis of Sporozoites

Details of the dissection of parous biting midges and staining methods were described by Valkiūnas [5] and Žiegytė et al. [29,30]. In brief, preparations of sporozoites were made after the extraction of the salivary glands from parous midges. The salivary glands were gently pressed out from the thorax, crushed using a needle and mixed with a tiny drop of saline. Preparations were dried in the air, fixed with absolute methanol, and stained with a 4% Giemsa stain. All residual parts of the midges were placed in 96% ethanol for PCR-based confirmation of parasite genetic lineages and insect species (as described below). To eliminate contamination of samples, we used a new dissecting needle for each dissected biting midge. Representative preparations of the sporozoites (49404–49408NS) were deposited in the Nature Research Centre, Vilnius, Lithuania. The statistical analyses of the parasite sporogonic stages were carried out using the “Statistica 7” package. Student’s t-test for independent samples was used to determine statistical significance between mean parameters of sporozoite features. A *p*-value < 0.05 was considered significant. 

### 2.3. Molecular Analysis

DNA from the remnants of each individual parous *Culicoides* female was extracted using an ammonium acetate DNA extraction method [31]. For the detection of avian haemosporidian parasites within insects, we used the nested PCR protocol described by [32,33,34] with outer primers HaemNFI/HaemNR3 and inner primers HaemF/HaemR2. A fragment of the mitochondrial cytochrome *b* (*cyt b*) gene (479 bp) of *Haemoproteus* and/or *Plasmodium* spp. was amplified. In order to detect false positives, we used a negative control (H2O instead of the target DNA) every 24 samples. To confirm the morphological identification of the *Culicoides* midges that contained haemospordian parasites, molecular analysis of the standard mitochondrial DNA cytochrome c oxidase subunit 1 (*cox1*) with primers LCO1490 and HCO2198 was applied [35]. DNA fragments of all samples were visualized on 2% agarose gel using MidoriGreen dye (NIPPON Genetics Europe, Germany). All positive samples were sequenced using forward and reverse primers. Sequences were edited and aligned using BioEdit software [36]. Genetic lineages of parasites were identified using the ‘Basic Local Alignment Search Tool’ (megablast algorithm) (NCBI BLAST, 2019 https://blast.ncbi.nlm.nih.gov/Blast.cgi, accessed on 1 March 2022), and their identification was double checked using the MalAvi database BLAST function (http://mbio-serv2.mbioekol.lu.se/Malavi, accessed on 1 March 2022).

## 3. Results

We collected 420 parous *Culicoides* females belonging to 10 species. The morphological identification was consistent with PCR-based identification of biting midges, obtained sequences matched corresponding sequences from GenBank 99–100%. *Culicoides kibunensis* was the dominant species and accounted for 30.4% of all collected parous biting midges (Table 1). DNA of *Haemoproteus* parasites of eight genetic lineages (haplotypes of the mitochondrial *cyt b* gene fragment) was detected in 28 *Culicoides* biting midges using PCR-based methods with the prevalence ranging from 0 to 20.8% for different *Culicoides* species (Table 1). The females of six *Culicoides* species were found to contain haemoproteid DNA: *C. kibunensis*, *C. pictipennis*, *C. festivipennis*, *C. segnis*, *C. obsoletus*, and *C. pallidicornis* (Table 1). The DNA of *Haemoproteus* was found for the first time in *C. pallidicornis* biting midges.

**Figure 2:**
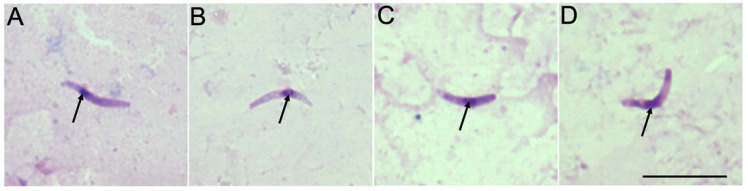
Sporozoites of *Haemoproteus majoris* (**A**) and *Haemoproteus tartakovskyi* (**B**) in salivary gland preparations of *Culicoides segnis*, and sporozoites of *Haemoproteus* *parabelopolskyi* (**C**) and *Haemoproteus* sp. (**D**) in salivary gland preparations of *Culicoides* *pictipennis*. Arrows indicate nuclei of the parasites. Scale-bar: 10 μm.

*Haemoproteus* sporozoites were detected in two salivary gland preparations of *C. segnis* biting midges, and PCR confirmed these parasites as being *H. majoris* (hCCF5) (Figure 2A) and *H. tartakovskyi* (hHAWF1) (Figure 2B). The sporozoites of *Haemoproteus* parasites were also detected in the salivary gland preparations of four *C. pictipennis* biting midges using microscopy. Three out of four midges were confirmed by PCR to be infected with *H. parabelopolskyi*, genetic lineage hSYAT02 (Figure 2C), while PCR failed to detect the parasite in one female insect (Figure 2D).

The results of the morphometrical analysis (Table 2) revealed that the length of *H. tartakovskyi* sporozoites differed statistically from the sporozoites of *H. majoris* (t = 2.41, *p* = 0.02) and *H. parabeloposkyi* (t = 4.99, *p* = 0.00), while the sporozoite width did not differ between different parasite species (*p* > 0.05). 

## 4. Discussion

Ten *Culicoides* species were identified from 420 investigated parous biting midge females, and *Culicoides kibunensis* was found to be the dominant species at the study site (Table 1). This species is also known to be among the dominant *Culicoides* species at other localities in Lithuania in June [21,37]. The composition of the *Culicoides* species on the Curonian Spit has been investigated by different authors in the southern part of the spit, which belongs to Russia [21,27,38,39]; however, this is the first study of *Culicoides* biting midges from the northern part of the Curonian spit, and it shows that the composition of the *Culicoides* species in different parts of the Curonian spit is similar. However, two new species for the Curonian spit (*C. chiopterus* and *C. fagineus*) were detected.

According to PCR-based data, 12 *Culicoides* species are known to harbor *Haemoproteus* parasite DNA in Europe, showing that biting midges of these species naturally feed on bird blood: *Culicoides alazanicus* [40], *C. circumscriptus* [41], *C. festivipennis* [24,40,42], *C. impunctatus* [23,24], *C. kibunensis* [21,24,43,44], *C. obsoletus* [24], *C. pictipennis* [21,24,40,44], *C. punctatus* [21,23,24], *C. segnis* [21,43], *C. scoticus* [24,44], *C. paolae* [41], and *C. reconditus* [21]. We have added *C. pallidicornis* to the list of *Culicoides* midges that feed naturally on birds and can be a potential vector of avian blood parasites. Previously, *C. pallidicornis* was attributed to mammalophilic species, as it preferentially feeds on cows, sheep [45], and/or rabbits [46].

The detection of haemosporidian DNA in biting midges is helpful in determining the host preference of the insects, as these parasites can be gained only during a bloodmeal on birds [23]. *Culicoides kibunensis* and *C. pictipennis* biting midges have been reported to feed preferentially on birds [46,47], and our results obtained using PCR have shown that the prevalence of haemoproteids in the examined *Culicoides* females of these species was relatively high (Table 1), showing that the ornithophily of *C. kibunensis*, *C. pictipennis,* and *C. segnis* is not a coincidence but a pattern. *Culicoides kibunensis* is known as a vector of *Haemoproteus pallidus* (hPFC1), *H. minutus* (hTURDUS2), and *H. asymmetricus* (hTUPHI01) [21,24]. Our study shows that the ornithophilic species *C. pictipennis* and *C. segnis* are also vectors of avian *Haemoproteus* parasites. 

To date, only sporadic cases of ornithophily of some other biting midges have been reported [45,48]. Currently, *C. punctatus* and *C. chiopterus* are known to feed on mammals [45,47,48,49], and we did not detect haemosporidian DNA in these biting midges during this study (Table 1), even though biting midges of these species accounted for more than 7% of all tested parous *Culicoides* females. *Culicoides impunctatus* is one of the most abundant *Culicoides* species in North Europe [1,38]: it was also abundant at our study site, accounting for 11% of all tested insects. It is known as being a mainly mammalophilic species. However, it was proved experimentally [30] that *C. impunctatus* can serve as a vector of 12 species of *Haemoproteus* parasites and can even be an opportunistic feeder on birds; thus, due to its high abundance, it can play an important role as a vector of haemoproteids.

The most important result of this study was that *H. majoris* (hCCF5) (Figure 2A) and *H. tartakovskyi* (hHAWF1) (Figure 2B) completed sporogony in *C. segnis*, and *H. parabelopolskyi* (hSYAT02) (Figure 2C) completed sporogony in *C. pictipennis* biting midges, showing that these blood-sucking insects are natural vectors of these haemosporidian parasites. The detection of both sporozoites and the PCR identification of the parasite in the same individual insect allowed us to indicate vectors of avian haemosporidian parasites in the wild and to obtain information about the specific genetic lineages of the detected parasites. It is known that sporogony of different *Haemoproteus* species with recorded sporozoite stages in salivary glands can be completed in four European *Culicoides* species: *C. impunctatus*, *C. nubeculosus*, *C. kibunensis,* and *C. sphagnumensis* [5,6,21,24,50]. We have added two new species, *C. segnis* and *C. pictipennis*, to the list of haemoproteid vectors.

*Haemoproteus parabelopolskyi* completed sporogony in two biting midge species: *C. impunctatus* [5,51,52] and *C. pictipennis* (this study). Valkiūnas et al. [51] recorded the complete sporogony of this parasite in *C. impunctatus* without determining the genetic lineage of the parasite. *Haemoproteus parabelopolskyi* is widespread and prevalent in warblers belonging to the Sylviidae, and this is the first species of hemosporidian parasite that has been described by linking molecular data and parasite morphology [52]. We revealed that the hSYAT02 lineage of *H. parabelopolskyi* completed its development in *C. pictipennis*. According to the available data, *H. tartakovskyi* completed sporogony and produced sporozoites in three species of biting midges: *C. impunctatus* [53], *C. nubeculosus* [48], and *C. segnis* (this study). Two previous studies showed the development of sporozoites after experimental infection of insects, while our study proved *C. segnis* as a vector of *H. tartakovskyi* in the wild. *Haemoproteus tartakovskyi* (hHAWF1) has been detected in wild caught biting midges for the first time. This parasite is widespread in passerine birds in the Palearctic with the common crossbill *Loxia curvirostra* as the type vertebrate host [5]. Additional vertebrate hosts of *H. tartakovskyi* are hawfinch *Coccothraustes* and Eurasian siskin *Spinus*. Heavy parasitemia of this parasite causes mortality in blood-sucking mosquitoes [19]. The sporozoites of *H. majoris* were detected in *C. impunctatus* after experimental infection of the insects [30]. Our study showed that *C. segnis* serves as a natural vector of *H. majoris* at the study site. *Haemoproteus majoris* is a widespread and prevalent parasite of different species, especially belonging to the families Paridae, Phylloscopidae, Fringillidae, and Muscicapidae [54].

It is necessary to emphasize that in some cases PCR-based methods may not detect the DNA of the parasite in insects, as was the case in this study (Figure 2D). The issue might be related to the low concentration of parasite DNA or to the specificity of the primers [55]. Therefore, in studies of haemosporidian vectors, it is important to use both methods, PCR and microscopy, in parallel.

Morphometric measurements of the length and width of the *H. tartakovskyi* sporozoites obtained from *C. segnis* (this study) and those provided by Žiegytė et al. [48] from experimentally infected *C. nubeculosus* did not differ significantly. Bukauskaite et al. [56] also stated that sporozoite measurements of the same parasite species (*H. noctue*) found in females of different *Culicoides* species did not differ significantly. However, the *Haemoproteus majoris* sporozoites detected in *C. segnis* during this study were shorter than those found in experimentally infected *C. impunctatus* biting midges (8.1 ± 0.5 and 9.5 ± 1.5 respectively, t = 4.09, *p* = 0.00) as described in Žiegytė et al. [30]. More comparative studies on the morphometric measurements of sporozoites obtained from different vectors are needed.

The diversity of *Culicoides* midges in Europe is high with more than 100 recorded species [3]. At the same time, more than 100 *Haemoproteus* species have been detected in birds. However, our knowledge about the transmission of *Haemoproteus* parasites is limited to a few *Culicoides* species that serve as vectors. This study adds to the knowledge of the epizootiology of haemoproteosis by revealing *Culicoides* species that are responsible for the transmission of haemoproteids in Europe and emphasizes obstacles in vector research.

## Figures and Tables

**Table 1:** Species list of collected and studied *Culicoides* females and detected haemoproteids.

*Culicoides* Species	No. of Investigated Parous Biting Midges	Prevalence (%)	Genetic Lineage of Parasite	Parasite Species (no. of Infected Individuals)
*C. kibunensis*	128	7.8	hWW1	*Haemoproteus palloris* (7)
			hWW2	*Haemoproteus majoris* (2)
			hPHYBOR04	*Haemoproteus majoris* (1)
*C. festivipennis*	58	3.5	hSYAT02	*Haemoproteus parabelopolskyi* (1)
			hHAWF1	*Haemoproteus tartakovskyi* (1)
*C. obsoletus*	50	6.0	hHAWF1	*Haemoproteus tartakovskyi* (2)
			hTUPHI01	*Haemoproteus asymmetricus* (1)
*C. impunctatus*	46	0		
*C. pictipennis*	37	18.9	hPARUS1	*Haemoproteus majoris* (1)
			hTUPHI01	*Haemoproteus asymmetricus* (3)
			hSYAT02	***Haemoproteus parabelopolskyi*** (3)
*C. punctatus*	36	0		
*C. chiopterus*	30	0		
*C. segnis*	24	20.8	hTUPHI01	*Haemoproteus asymmetricus* (1)
			hCCF5	***Haemoproteus majoris*** (2)
			hHAWF1	***Haemoproteus tartakovskyi*** (2)
*C. pallidicornis*	9	11.1	hWW1	*Haemoproteus palloris* (1)
*C. fagineus*	2	0		

*Haemoproteus* species of which sporozoites were detected in salivary glands are in bold.

**Table 2:** Morphometric parameters of sporozoites of three *Haemoproteus* species.

*Haemoproteus* Species (no. of Examined Sporozoites)	Length (min–max)	Width (min–max)	Area (min–max)
*H. parabeloposkyi* * (21)	7.2 ± 1.01 (5.5–8.8)	1.1 ± 0.09 (0.9–1.2)	6.3 ± 0.9 (4.5–7.9)
*H. majoris* ** (21)	8.1 ± 0.47 (7.2–9.0)	1.1 ± 0.15 (0.9–1.4)	7.2 ± 0.69 (5.2–8.5)
*H. tartakovskyi* ** (21)	8.5 ± 0.55 (7.8–9.5)	1.1 ± 0.16 (0.8–1.5)	7.1 ± 0.89 (5.5–8.8)

*Haemoproteus* parasites detected in *C. pictipennis* (*), and in *C. segnis* (**). Measurements are given in micrometers. Arithmetic mean and standard deviation are provided, followed in parentheses by minimum and maximum values.

## Data Availability

The data presented in this study are available upon email inquiry.

## References

[1] Carpenter S., Groschup M.H., Garros C., Felippe-Bauer M.L., Purse B. (2013). *Culicoides* biting midges, arboviruses and public health in Europe. Antivir. Res..

[2] Borkent A. World Species of Biting Midges (Diptera: Ceratopogonidae). https://digitallibrary.amnh.org/handle/2246/1622.

[3] Borkent A., Dominiak P. (2020). Catalog of the Biting Midges of the World (Diptera: Ceratopogonidae). Zootaxa.

[4] Wirth W. (1977). A Review of the Pathogens and Parasites of the Biting Midges (Diptera: Ceratopogonidae). J. Wash. Acad. Sci..

[5] Valkiūnas G. (2005). Avian Malaria Parasites and Other Haemosporidia.

[6] Atkinson C.T., Thomas N.J., Hunter D.B. (2008). Parasitic Diseases of Wild Birds.

[7] Atkinson C.T. (1991). Vectors, epizootiology, and pathogenicity of avian species of *Haemoproteus* (Haemosporina: Haemoproteidae). Bull. Soc. Vector Ecol..

[8] Santiago-Alarcon D., Palinauskas V., Schaefer H.M. (2012). Diptera vectors of avian haemosporidian parasites: Untangling parasite life cycles and their taxonomy. Biol. Rev..

[9] Santiago-Alarcon D., Marzal A. (2020). Avian Malaria and Related Parasites in the Tropics.

[10] Garnham P.C.C. (1966). Malaria Parasites and Other Haemosproridia.

[11] Atkinson C.T., Forrester D.J., Greiner E.C. (1988). Pathogenicity of *Haemoproteus meleagridis* (Haemosporina: Haemoproteidae) in experimentally infected domestic turkeys. J. Parasitol..

[12] Earleé R.A., Bastianello S.S., Bennett G.F., Krecek R.C. (1993). Histopathology and morphology of the tissue stages of *Haemoproteus columbae* causing mortality in Columbiformes. Avian Pathol..

[13] Donovan T.A., Schrenzel M., Tucker T.A., Pessier A.P., Stalis I.H. (2008). Hepatic hemorrhage, hemocoelom, and sudden death due to *Haemoproteus* infection in passerine birds: Eleven cases. J. Vet. Diagn. Investig..

[14] Olias P.M., Wegelin W., Zenker S., Freter A., Gruber D., Klopfleisch R. (2011). Avian malaria deaths in parrots. Eur. Emerg. Infect. Dis..

[15] Cannell B.L., Krasnec K.V., Campbell K., Jones H.I., Miller R.D., Stephens N. (2013). The pathology and pathogenicity of a novel *Haemoproteus* spp. infection in wild little penguins (*Eudyptula minor*). Vet. Parasitol..

[16] Tostes R., Martinele I., Vashist U., Castanñon M.C., Pinto P.F., Daemon E., D’Agosto M. (2015). Molecular characterization and biochemical and histopathological aspects of the parasitism of *Haemoproteus* spp. in southern caracaras (*Caracara plancus*). J. Parasitol..

[17] Ortiz-Catedral L., Brunton D., Stidworth M.F., Elsheikha H.M., Pennycott T., Schulze C., Braun M., Wink M., Gerlach H., Pendl H. (2019). *Haemoproteus minutus* is highly virulent for Australasian and South American parrots. Parasit. Vectors.

[18] Hernández-Lara C., Duc M., Ilgūnas M., Valkiūnas G. (2021). Massive Infection of Lungs with Exo-Erythrocytic Meronts in European Robin *Erithacus rubecula* during Natural *Haemoproteus attenuatus* Haemoproteosis. Animals.

[19] Valkiūnas G., Kazlauskienė R., Bernotienė R., Bukauskaitė D., Palinauskas V., Ježova T. (2014). *Haemoproteus* infections (Haemosporida, Haemoproteidae) kill bird-biting mosquitoes. Parasitol. Res..

[20] Bukauskaitė D., Bernotienė R., Iezhova T.A., Valkiūnas G. (2016). Mechanisms of mortality in *Culicoides* biting midges due to *Haemoproteus* infection. Parasitology.

[21] Žiegytė R., Platonova E., Kinderis E., Mukhin A., Palinauskas V., Bernotienė R. (2021). *Culicoides* biting midges involved in transmission of haemoproteids. Parasit. Vectors.

[22] Valkiūnas G., Kazlauskienė R., Bernotienė R., Palinauskas V., Iezhova T.A. (2013). Abortive long-lasting sporogony of two *Haemoproteus* species (Haemosporida, Haemoproteidae) in the mosquito *Ochlerotatus cantans*, with perspectives on haemosporidian vector research. Parasitol. Res..

[23] Bernotienė R., Valkiūnas G. (2016). PCR detection of malaria parasites and related haemosporidians: The sensitive methodology in determining bird-biting insects. Malar. J..

[24] Bernotienė R., Žiegytė R., Vaitkutė G., Valkiūnas G. (2019). Identification of a new vector species of avian haemoproteids, with a description of methodology for the determination of natural vectors of haemosporidian parasites. Parasit. Vectors.

[25] Dyce A.L. (1969). The recognition of nulliparous and parous *Culicoides* (Diptera: Ceratopogonidae) without dissection. Aust. J. Entomol..

[26] Gutsevich A.V. (1973). Blood-sucking midges (Ceratopogonidae). Fauna of the USSR.

[27] Glukhova V.M. (1989). Blood-sucking midges of the genera *Culicoides* and *Forcipomyia* (Ceratopogonidae). Fauna of the USSR. Dipteran Insects.

[28] Mathieu B., Ceêtre-Sossah C., Garros C., Chavernac D., Balenghien T., Carpenter S., Setier-Rio M.L., Vignes-Lebbe R., Ung V., Candolfi E. (2012). Development and validation of IIKC: An interactive identification key for *Culicoides* (Diptera: Ceratopogonidae) females from the Western Palaearctic region. Parasit. Vectors.

[29] Žiegyteė R., Palinauskas V., Bernotienė R., Iezhova T.A., Valkiūnas G. (2014). *Haemoproteus minutus* and *Haemoproteus belopolskyi* (Haemoproteidae): Complete sporogony in the biting midge *Culicoides impunctatus* (Ceratopogonidae), with implications on epidemiology of Haemoproteosis. Exp. Parasitol..

[30] Žiegytė R., Markovets M.Y., Bernotienė R., Mukhin A., Iezhova T.A., Valkiūnas G., Palinauskas V. (2017). The widespread biting midge *Culicoides impunctatus* (Ceratopogonidae) is susceptible to infection with numerous *Haemoproteus* (Haemoproteidae) species. Parasit. Vectors.

[31] Richardson D.S., Jury F.L., Blaakmeer K., Komdeur J., Burke T. (2001). Parentage assignment and extra group paternity in a cooperative breeder: The Seychelles warbler (*Acrocephalus sechellensis*). Mol. Ecol..

[32] Bensch S., Stjenman M., Hasselquist D., Ostman O., Hansson B., Westerdahl H., Pinheiro R.T. (2000). Host specificity in avian blood parasites: A study of *Plasmodium* and *Haemoproteus* mitochondrial DNA amplified from birds. Proc. R. Soc..

[33] Hellgren O., Waldenstrom J., Bensch S. (2004). A new PCR assay for simultaneous studies of *Leucocytozoon*, *Plasmodium*, and *Haemoproteus* from avian blood. J. Parasitol..

[34] Hellgren O., Bensch S., Malmqvist B. (2008). Bird hosts, blood parasites and their vectors-associations uncovered by molecular analyses of blackfly blood meals. Mol. Ecol..

[35] Folmer O., Black M., Hoeh W., Lutz R., Vrijenhoek R. (1994). DNA primers for amplification of mitochondrial cytochrome *c* oxidase subunit I from diverse metazoan invertebrates. Mol. Mar. Biol. Biotechnol..

[36] Hall T.A. (1999). A user-friendly biological sequence alignment editor and analysis program for Windows 98/98/NT. Nucleic. Acid. Symp. Ser..

[37] Bernotienė R., Bartkevičienė G., Bukauskaitė D. (2021). The flying activity of biting midges (Ceratopogonidae: *Culicoides*) in Verkiai Regional Park, southeastern Lithuania. Parasitol. Res..

[38] Glukhova V.M., Valkiūnas G. (1993). On the fauna and ecology of biting midges (Ceratopogonidae: *Culicoides*) in the Curonian spit, the methods of their collection from the birds and experimental infection with haemoproteids (Haemosporidia: Haemoproteidae). Ekologija.

[39] Trukhan M.N., Tereshkina N.V., Liutkevičius G. (2003). Peculiarities of the range of species and the ecology of midges (Diptera, Ceratopogonidae) on the Curonian spit. Vesci Nacyanalnaj Akad. Navuk Belarusi.

[40] Bobeva A., Zehtindjiev P., Bensch S., Radrova J. (2013). A survey of biting midges of the genus *Culicoides* Latreille, 1809 (Diptera: Ceratopogonidae) in NE Bulgaria, with respect to transmission of avian haemosporidians. Acta Parasitol..

[41] Veiga J., Martinez-de la Pueante J., Vaclav R., Figuerola J., Valera F. (2018). *Culicoides paolae* and *C. circumscriptus* as potential vectors of avian haemosporidians in an arid ecosystem. Parasit. Vectors.

[42] Bobeva A., Ilieva M., Dimitrov D., Zehtindjiev P. (2014). Degree of associations among vectors of the genus *Culicoides* (Diptera: Ceratopogonidae) and host bird species with respect to haemosporidian parasites in NE Bulgaria. Parasitol. Res..

[43] Synek P., Munclinger P., Albrecht T., Votýpka J. (2013). Avian haematophagous insects in the Czech Republic. Parasitol. Res..

[44] Santiago-Alarcόn D., Havelka P., Pineda E., Segelbacher G., Schaefer H.M. (2013). Urban forests as hubs for novel zoonosis: Blood meal analysis, seasonal variation in *Culicoides* (Diptera: Ceratopogonidae) vectors, and avian haemosporidians. Parasitology.

[45] Aylloón T., Nijhof A.M., Weiher W., Bauer B., Alleène X., Clausen P.H. (2014). Feeding behaviour of *Culicoides* spp. (Diptera: Ceratopogonidae) on cattle and sheep in northeast Germany. Parasit. Vectors.

[46] Ninio C., Augot D., Delecolle J.C., Dufour B., Depaquit J. (2010). Contribution to the knowledge of *Culicoides* (Diptera: Ceratopogonidae) host preferences in France. Parasitol. Res..

[47] Miltgen F., Landau I., Ratanaworabhan N., Yenbutra S. (1981). *Parahaemoproteus desseri* n. sp.; Gametogonie et shizogonie chez I’hote naturel: *Psittacula roseate* de Thailande, et sporogonie experimentale chez *Culicoides nubeculosus*. Ann. Parasitol. Hum. Comp..

[48] Žiegytė R., Bernotienė R., Palinauskas V., Valkiūnas G. (2016). *Haemoproteus tartakovskyi* (Haemoproteidae): Complete sporogony in *Culicoides nubeculosus* (Ceratopogonidae), with implications for avian haemoproteid experimental research. Exp. Parasitol..

[49] Lassen S.B., Nielsen S.A., Skovgård H., Kristensen M. (2011). Molecular identification of bloodmeals from biting midges (Diptera: Ceratopogonidae: *Culicoides* Latreille) in Denmark. Parasitol. Res..

[50] Bukauskaitė D., Iezhova T.A., Ilgūnas M., Valkiūnas G. (2019). High susceptibility of the laboratory-reared biting midges *Culicoides nubeculosus* to *Haemoproteus* infections, with review on *Culicoides* species that transmit avian haemoproteids. Parasitology.

[51] Valkiūnas G., Iezhova T.A. (2004). Detrimental effects of *Haemoproteus* infections on the survival of biting midge *Culicoides impunctatus* (Diptera: Ceratopogonidae). J. Parasitol..

[52] Valkiūnas G., Križanauskienė A., Iezhova T.A., Hellgren O., Bensch S. (2007). Molecular phylogenetic analysis of Circumnuclear hemoproteids (Haemosporida: Haemoproteidae) of sylviid birds, with a description of *Haemoproteus parabelopolskyi* sp. Nov. Parasitol..

[53] Valkiūnas G., Liutkevičius G., Iezhova T.A. (2002). Complete development of three species of *Haemoproteus* (Haemosporida, Haemoproteidae) in the biting midge *Culicoides impunctatus* (Diptera, Ceratopogonidae). J. Parasitol..

[54] Bensch S., Hellgren O., Pérez-Tris J. (2009). MalAvi: A public database of malaria parasites and related haemosporidians in avian hosts based on mitochondrial cytochrome *b* lineages. Mol. Ecol. Resour..

[55] Harl J., Himmel T., Valkiūnas G., Ilgūnas M., Nedorost N., Matt J., Kubber-Heiss A., Alic A., Konicek C., Weissenbock H. (2022). Avian haemosporidian parasites of accipitriform raptors. Malar. J..

[56] Bukauskaitė D., Žiegytė R., Palinauskas V., Iezhova T.A., Dimitrov D., Ilgūnas M., Bernotienė R., Markovets M.Y., Valkiūnas G. (2015). Biting midges (*Culicoides*, Diptera) transmit *Haemoproteus* parasites of owls: Evidence from sporogony and molecular phylogeny. Parasit. Vectors.

